# Supplementary material for: Self-heating hotspots in superconducting nanowires cooled by phonon black-body radiation
Source: Nat Commun. 2022 Sep 16;13:5429. doi: 10.1038/s41467-022-32719-w (PMC9481646; doi:10.1038/s41467-022-32719-w)
Supplement: Supplementary file 1 — Supplementary Information [file 41467_2022_32719_MOESM1_ESM.pdf]

Supplementary Information for "Self-Heating Hotspots in Superconducting Nanowires Cooled by Phonon Black-Body Radiation"

Index:

1. Supplementary Methods: Details of our one-dimensional electro-thermal simulation
2. Supplementary Tables and Discussion: Recreating Table II from Swartz and Pohl 1989
3. Supplementary Tables: Calculated $\sigma_{ph}\epsilon_{AMM}$ for select substrate-superconductor pairs
4. Supplementary Figure: Calculated $\sigma_{ph}\epsilon_{AMM}$ for select substrate-superconductor pairs
5. Supplementary Figures: All $I_{hs}(T_b)$ data and fits
6. Supplementary Table: Summary Comparing β to $\sigma_{ph}\epsilon_{AMM}$ and $\sigma_{ph}\epsilon_{DMM}$
7. Supplementary Note: Our re-analysis of Kadin's data
8. Supplementary Note: Excluding the Nb-Ti bilayer devices
9. Supplementary References

Supplementary Methods: details of our one-dimensional electro-thermal simulation

To study the validity of the approach discussed in the main text, we numerically simulated the hotspot current of thin film nanowires in a variety of conditions. To simplify the analysis we used the quasi-equilibrium two-temperature model to describe the energy flow within the nanowire, solved in a 1D geometry. While the true stationary state of the system contains a non-equilibrium distribution of phonons in the thin film, we neglected the details of these distribution functions. Under these simplifications, the energy balance equation for the electron system is given by:

$$C_e(T_e)\frac{\partial T_e}{\partial t} = -\Sigma_{e-ph}\left(T_e^5 - T_{ph}^5\right) + \nabla\kappa_e(T_e)\nabla T_e + j^2\rho(T_e, j) \quad (1)$$

while the phonon system is described by:

$$C_{ph}\left(T_{ph}\right)\frac{\partial T_{ph}}{\partial t} = \Sigma_{e-ph}\left(T_e^5 - T_{ph}^5\right) + \nabla\kappa_{ph}\left(T_{ph}\right)\nabla T_{ph} - \beta\left(T_{ph}^4 - T_b^4\right) \quad (2)$$

In (1) and (2), T_e is the electron temperature, T_{ph} is the phonon temperature, T_b is the bath temperature, C_e is the electron heat capacity, Σ_{e-ph} is the electron-phonon coupling constant, κ_e is the electron thermal conductivity, j is the current density, ρ is the resistivity, C_{ph} is the phonon heat capacity, κ_{ph} is the phonon thermal conductivity, and β describes the thermal boundary conductance between the nanowire and substrate as discussed in the main text. The current density is defined as $j = \frac{I_B}{(w \cdot d)}$ for the bias current I_B, nanowire width w, and thickness d. The electron thermal conductivity is given by the Bardeen equation [3]:

$$k_e = \frac{2D\pi^2 k_B^2 N(0) T_e}{3}\left(1 - \frac{6}{\pi^2}\int_0^{|\Delta|/k_B T_e} \frac{x^2 e^x dx}{(e^x + 1)^2}\right) \quad (3)$$

where the magnitude of the order parameter $|\Delta|$ is evaluated as the zero current BCS order parameter at the temperature T_e. In (3), D is the diffusion coefficient, k_B is the Boltzmann constant, and $N(0)$ is the single-spin electron density of states in the normal state, at the Fermi level. The

electron heat capacity is given by the BCS heat capacity, and the electron-phonon coupling constant is given by the expression $\Sigma_{e-ph} = \frac{96\zeta(5)N(0)k_B^2}{T_c^3\tau_0}$ where T_c is the critical temperature, and τ_0 is a characteristic electron-phonon coupling time [4][5]. The fifth power form of the electron-phonon coupling term in Eqn. (2) assumes that the electron system is in the normal state, which is reasonable given that the region of interest is the normal domain within the nanowire. The phonon heat capacity is given by the Debye model and the phonon thermal conductivity is calculated by assuming a phonon mean free path that is limited by the film thickness (the Casimir limit):

$$k_{ph}(T_{ph}) = \frac{v_{avg}d}{3}C_{ph}(T_{ph}) \tag{4}$$

where v_{avg} is the mode-averaged sound velocity of the material. This also defines the phonon diffusion coefficient $D_{ph} = \frac{v_{avg}d}{3}$.

The transition between the superconducting and normal states is handled phenomenologically through the temperature and current density dependent resistivity function $\rho(T_e, j)$. The resistivity function is defined as:

$$\rho(T_e, j) = \frac{R_s d}{1 + e^{\frac{T_c(j) - T_e}{\sigma_T}}} \tag{5}$$

The current density dependent critical temperature $T_c(j)$ is defined as

$$\frac{T_c(j)}{T_{c0}} \approx \left(1 - \left(\frac{j}{j_{dep}(0)}\right)^{\frac{2}{3}}\right)^{\frac{1}{2}} \tag{6}$$

Which uses the Bardeen temperature dependence of the critical current combined with the zero-temperature critical depairing current density $j_{dep}(0)$ calculated from the solution of the Usadel equations [6]. In (5), R_s is the sheet resistance, σ_T is the width of the resistive transition in Kelvin.

The energy balance equations are solved together with the circuit equation

$$V_{bias} = (R_{bias} + R_{hs})I_{bias} + L_k \frac{dI_{bias}}{dt} \tag{7}$$

which describes the response of the nanowire in series with a voltage source V_{bias}, bias resistor R_{bias}, and inductance L_k, which corresponds to the circuit used to experimentally probe the hotspot current. The resistance of the nanowire R_{hs} is given by integrating over the length of the simulated nanowire:

$$R_{hs} = \frac{1}{wd}\int_{-L/2}^{L/2} \rho(T_e(x), j)\, dx \tag{8}$$

where L is the length of the nanowire.

Solutions to our 1D electrothermal equations depend on four characteristic parameters, three time scales, and one length scale, and are listed here with comments. The time scales in our electrothermal equations were normalized by $\frac{\hbar}{k_B T_c}$, making them unitless. Similarly, temperatures were normalized by T_c, and current by $j_{dep}(0)$. The characteristic length scale is set by electron diffusion at the critical temperature. For the range of parameters representative of the films characterized in this work, the phonon diffusion term is small compared to the electron diffusion and can be neglected $\left(\frac{D_{ph}}{D} \ll 1\right)$. Under this simplification, the stationary state of the self-heating hotspot depends on only four characteristic parameters:

$$\tilde{\tau}_0 = \frac{\tau_0}{\hbar/k_B T_c}, \quad \tilde{x} = \sqrt{\frac{\hbar D}{k_B T_c}}, \quad \gamma_{ph}\tilde{\tau}_{esc} = \frac{\gamma_{ph}\tau_{esc}}{\hbar/k_B T_c}, \quad \tilde{T}_b = \frac{T_b}{T_c}$$

Here we use the characteristic phonon parameter, $\gamma_{ph} = \frac{8\pi^2}{5}\frac{C_e}{C_{ph}}\Big|_{T=T_c}$ which describes the ratio of the heat capacities in the electron and phonon systems at T_c [5] and the phonon escape time, $\tau_{esc} = \frac{dC_{ph}(T_c)}{4\beta T_c^3}$. The parameter $\tilde{\tau}_0$ is a normalized version of the characteristic time introduced in [4].

The accuracy of the fitting procedure described in the main text can be evaluated for combinations of $\tilde{\tau}_0$, $\tilde{x}$, and $\gamma_{ph}\tilde{\tau}_{esc}$. Simulations were preformed numerically by nucleating a normal domain in the nanowire and allowing the system to evolve to a stationary state with a self-heating hotspot. Once a stable hotspot was formed, the hotspot current density was extracted. This process was repeated for various V_{bias} and T_b to generate a hotspot current density versus substrate temperature curve analogous to the ones measured experimentally. For all of these simulations, we use $\sigma_T = 0.005T_c$ and confirm that changing this parameter does not significantly alter the results. We varied T_b such that $\tilde{T}_B = 0.1 - 0.85$.

We fit the results with a function of the form

$$I_{hs}^2 \frac{R_s}{w^2} = \beta_{fit}(T_{hs}^4 - T_b^4) \tag{9}$$

Which is

$$\tilde{J}_{hs}^2 \frac{1.491^2 1.764^3}{8} \frac{15}{\pi^4} \gamma_{ph}\tilde{\tau}_{esc} = \frac{\beta_{fit}}{\beta_{sim}}\left(\tilde{T}_{hs}^4 - \tilde{T}_b^4\right) \tag{10}$$

in non-dimensional terms. The fitting parameter β_{fit}/β_{sim} is the ratio of the thermal boundary conductance determined by fitting to the true value used in the simulation. $\tilde{T}_{hs}$ is the extracted and normalized hotspot temperature. Thus β_{fit}/β_{sim} represents the accuracy with which the fitting procedure reproduces the results generated by the two-temperature electrothermal model.

In fig. S1, we plot the value of β_{fit}/β_{sim} resulting from performing our simulation and fitting procedure for a range of $\tilde{\tau}_0$, $\tilde{x}$, and $\gamma_{ph}\tilde{\tau}_{esc}$. While a region of parameter space exists where $\beta_{fit} \sim \beta_{sim}$, in general, the accuracy of this approach is material dependent. Using known material parameters, our calculations can be used to generate a correction factor which can be used to better approximate the true value of β based on the extracted fit value of the thermal boundary

conductance. For the range of $\tilde{x}$ relevant for typical SNSPD materials, $\tilde{x}$ has little influence on the accuracy of the fitting procedure as demonstrated by the nearly identical results for the three values shown.

Here, we use our simulation to examine the accuracy of our procedure for a few exemplar substrate types. If we take $T_c = 8.3\ K$ based on the experimental results, and scale $\tau_{ep}(10\ K) = 12 - 16\ ps$ [7] to 8.3 K based on an inverse cubic law for clean metals, we arrive at $\tilde{\tau}_0 = 1725 - 2480$. Using $D = 0.35 - 0.5\ {cm^2}/{s}$ leads to $\tilde{x} = 5.7 - 6.9\ nm$. Taking $R_s = 570\ \Omega$, based on NbN on sapphire, $d = 5\ nm$, and $v_{avg} = 5465\ {m}/{s}$ leads to $\gamma_{ph} \sim 50$. With these parameters, $\beta = 128\ {W}/{m^2K^4}$ which leads to $\tau_{esc} = 7.3\ ps$ and $\gamma_{ph}\tilde{\tau}_{esc} \sim 420$. This corresponds to a value of ${\beta_{fit}}/{\beta_{sim}} = 0.77 - 0.89$ based on the estimated range of $\tilde{\tau}_0$. Similarly, if we take representative values of the $LiNbO_3$ data with $R_s = 800\ \Omega$ and $\beta = 207\ {W}/{m^2K^4}$, we arrive at ${\beta_{fit}}/{\beta_{sim}} = 0.41 - 0.50$. Finally, if we consider samples on thermal oxide with $T_c = 7.7\ K$, $R_s = 1100\ \Omega$, and $\beta = 168\ {W}/{m^2K^4}$, we find ${\beta_{fit}}/{\beta_{sim}} = 0.37 - 0.45$ for the range of $\tilde{\tau}_0$ considered. These estimates are qualitatively consistent with experimental findings.

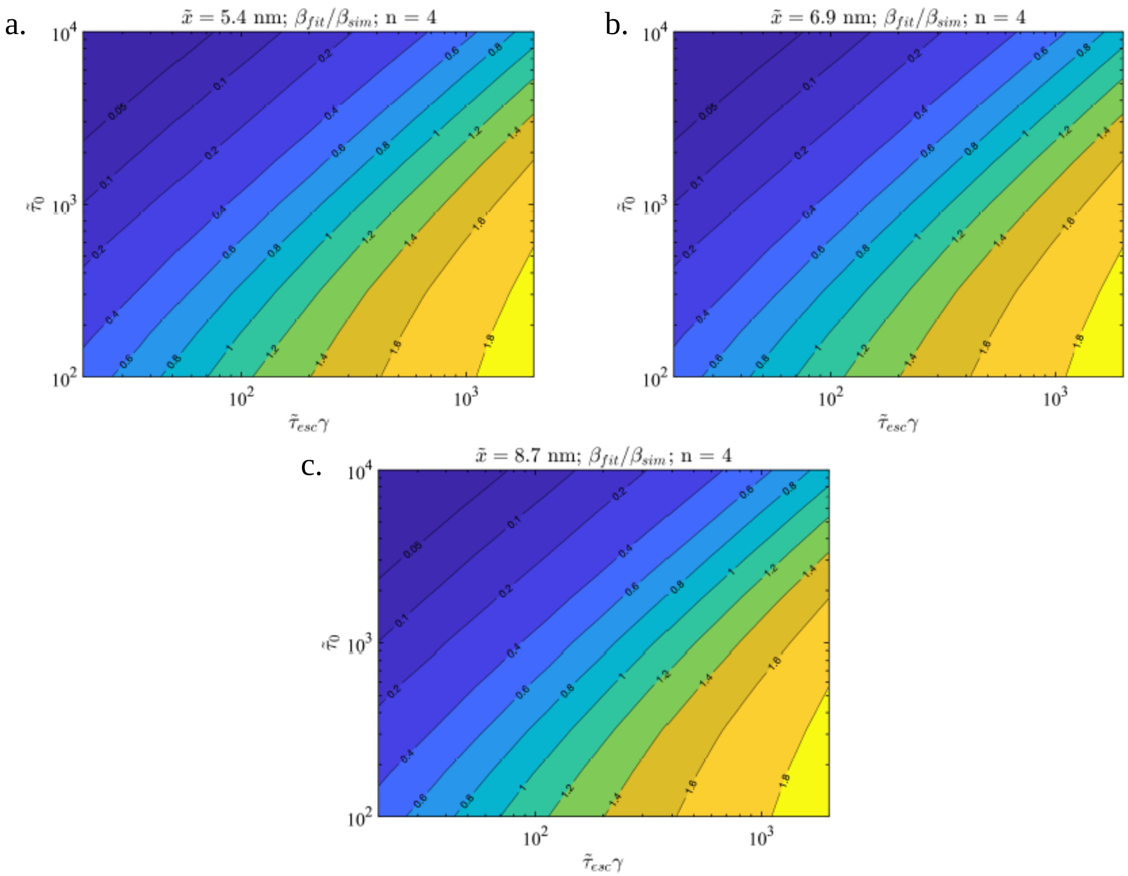

Supplementary Figure 1. Calculated β_{fit}/β_{sim} for a range of $\tilde{\tau}_0$ and $\gamma_{ph}\tilde{\tau}_{esc}$ with (a) $\tilde{x} = 5.4\,nm$, (b) $\tilde{x} = 6.9\,nm$, or (c) $\tilde{x} = 8.7\,nm$. Ideally the value extracted from the fit would reproduce the value entered into the simulation exactly, such that $\beta_{fit}/\beta_{sim} = 1$. By comparing panels, we can see that $\tilde{x}$ does not substantially affect the accuracy of the hotspot current fitting procedure.

Supplementary Tables and Discussion: recreating Table II from Swartz and Pohl 1989

In order to verify the accuracy of our AMM and DMM calculations, we reproduced some of the calculations given in Swartz and Pohl's 1989 review article, ref [8]. We used the material properties listed in Table S1, which is a subset of Table 1 in [8], to calculate the thermal boundary resistance between pairs of materials using the acoustic and diffuse mismatch models. Table S2 gives the results of our calculations. We also calculated the percent errorof our results from [8], by dividing each entry in Supplementary Table 2 with the corresponding entry from table II of [8], then subtracting one. The percent error of each entry is given in Table S3. The median error is 0%, however some AM entries exceed 10% deviation, and our results for diamond appear to have a systematically high error. It is worth noting that our values are calculated by one piece of software given the input material properties, whereas the entries in table II of [8] come from published lookup tables [9]. Deviations of up to 30% between the values reported in [9] and those calculated by Kaplan were previously reported [10].

Material:	Density (g/cm^3)	C_L (10^5 cm/sec)	C_T (10^5 cm/sec)
Aluminum	2.7	6.24	3.04
Chromium	7.19	6.98	4.1
Copper	8.96	4.91	2.5
Gold	19.3	3.39	1.29
Indium	7.47	2.699	0.905
Lead	11.59	2.35	0.97
Nickel	8.81	5.63	2.96
Platinum	21.62	4.174	1.75
Rhodium	12.4	5.83	3.96
Silver	10.63	3.78	1.74
Sapphire	3.97	10.89	6.45
Quartz	2.66	6.09	4.1
Silicon	2.33	8.97	5.332

Supplementary Table 1. Materials and their corresponding densities and speeds of sound, reproduced from Swartz and Pohl 1989. We used these values as inputs to our AMM and DMM calculations, in order to verify their accuracy.

Diamond	3.512	17.5	12.8
Calcite	2.717	6.75	3.48
CaF_2	3.217	6.92	3.69

To avoid potential confusion, we further explain how to translate between what we show in the main text, and the values we give in Table S2. The entries in table II of [8] are linearized thermal boundary *resistances* multiplied by T^3. In the main text we consider the thermal boundary conductance, without linearization. In the main text, we are primarily concerned with heat transfer of the form $Q = \sigma\epsilon w^2\left(T_{high}^4 - T^4\right)$. For any T and T_{high} we can write $T_{high} = T + \Delta T$. If $\Delta T \ll T$, and we substitute $T + \Delta T$ for T_{high}, expand terms, subtract T^4, and drop any terms that contain powers of ΔT greater than the first, we get $Q \approx 4\sigma\epsilon w^2 T^3 \Delta T$. This is an equation for the heat flow Q, which is linear in the temperature drop across the material boundary (ΔT). We can re-write this as $Q \approx w^2 \frac{\Delta T}{R_{Bd}}$ with $R_{Bd} = \frac{1}{4\sigma\epsilon T^3}$. R_{Bd} is the thermal boundary resistance as written in [8], with units Kcm^2W^{-1}. The entries in Table II of [8] are given as $R_{Bd}T^3$, which we can now see is simply equal to $\frac{1}{4\sigma\epsilon}$.

	Sapphire		Quartz		Silicon		Diamond		Calcite		CaF_2	
	AMM	DMM	AMM	DMM	AMM	DMM	AMM	DMM	AMM	DMM	AMM	DMM
Aluminum	21.1	21.4	7.2	10.8	12.7	15.9	96.9	67.5	5.4	9.3	6.1	9.9
Chromium	18.8	24.4	9.8	13.8	15.6	18.9	67.8	70.4	9.6	12.3	8.8	12.9
Copper	18.6	20.1	8.9	9.4	15.1	14.6	69.3	66.1	6.8	8.0	7.0	8.6
Gold	19.2	18.1	10.6	7.5	16.8	12.6	68.7	64.2	8.2	6.0	8.2	6.6
Indium	21.2	17.7	7.3	7.1	12.6	12.2	99.2	63.8	5.6	5.6	6.2	6.2
Lead	19.0	17.8	7.6	7.1	12.9	12.3	81.2	63.8	6.0	5.7	6.4	6.3
Nickel	18.8	21.1	9.2	10.5	15.7	15.6	68.3	67.2	7.4	9.0	7.4	9.6
Platinum	21.3	18.7	13.3	8.1	20.7	13.2	70.4	64.8	9.8	6.6	9.8	7.2
Rhodium	21.4	23.6	13.2	13.0	20.3	18.1	70.4	69.7	12.2	11.5	11.1	12.1
Silver	18.5	18.7	8.8	8.1	14.4	13.2	71.1	64.8	6.8	6.6	7.0	7.2

Supplementary Table 2. Linearized thermal boundary resistance for row-column pairs, using both the acoustic mismatch (AMM) and diffuse mismatch (DMM) models. This table is a recreation of Table II in [8], using our code.

The same code was used to predict the expected thermal boundary conductance that is mentioned in the main text. Each entry has units K^4cm^2/W.

	Sapphire		Quartz		Silicon		Diamond		Calcite		CaF_2	
	AMM	DMM	AMM	DMM	AMM	DMM	AMM	DMM	AMM	DMM	AMM	DMM
Aluminum	0%	0%	11%	0%	8%	0%	24%	0%	3%	0%	1%	0%
Chromium	2%	0%	0%	0%	8%	0%	13%	0%	15%	0%	7%	0%
Copper	0%	0%	3%	0%	5%	0%	13%	0%	1%	0%	1%	0%
Gold	2%	0%	31%	0%	6%	0%	14%	0%	3%	0%	7%	0%
Indium	4%	0%	1%	0%	4%	0%	13%	0%	2%	0%	0%	0%
Lead	1%	0%	0%	0%	1%	0%	8%	0%	2%	0%	1%	0%
Nickel	-4%	0%	-1%	0%	1%	0%	11%	0%	-12%	0%	-13%	0%
Platinum	3%	0%	2%	0%	-3%	0%	16%	0%	5%	0%	5%	0%
Rhodium	3%	0%	2%	0%	6%	0%	14%	0%	12%	0%	8%	0%
Silver	1%	0%	1%	0%	4%	0%	16%	0%	4%	0%	5%	0%

Supplementary Table 3. The percent difference between the entries in Table II of [8] and our recalculation (Table S2). The DMM values are based on a simple formula using the same inputs. As such, our recalculation matches exactly. On the other hand, the AMM values in Table II of [8] are based on lookup tables in ref [9], while ours were calculated as needed based on the acoustic properties of the materials. The nature of using lookup tables allows for the introduction of a variety of errors that our calculations would not be susceptible to. Discrepancies between the lookup table in [9] and the values calculated by Kaplan were reportedly up to 30% [10].

Supplementary Tables: calculated $\sigma_{ph}\epsilon_{AMM}$ for select substrate-superconductor pairs

We used material properties collected from literature as inputs to our AMM and DMM calculations, paying particular attention to materials and substrates that may be important for superconducting device applications. The material parameters used are given in Table S4, while the results are reported in Table S5. The materials properties are compiled from [10], [11], and [12]. In Table S5 we include the longitudinal (η_L) and transverse (η_T) transmission coefficients for comparison with Kaplan [10].

Material:	Density (g/cm^3)	c_L (km/s)	c_T (km/s)
Al	2.73	6.65	3.26
MoSi	9.2	6.1	2.7
Nb	8.59	5.14	2.17
NbN	8.3	8.77	4.91
TiN	5.4	7.8	4.503
WSi	14.2	5.5	4.5
AlN	3.23	10.13	6.33
Al_2O_3	3.99	10.9	6.45
GaN	6.15	7.96	4.13
LiF	7.32	4.52	2.64
$LiNbO_3$	4.65	7.43	3.715
MgO	3.59	9.68	6.06
PET	1.4	2.5	1.21
Si	2.33	8.98	5.34
SiC (3C)	3.21	9.5	4.1
a-SiN	2.9	10.3	6.2
SiO_2	2.66	6.09	4.09
$SrTiO_3$	5.11	7.87	4.9

Supplementary Table 4. Material properties from literature, used to calculate the thermal boundary conductance values given in Supplementary Table 5.

	Al				MoSi				Nb			
	η_L	η_T	AMM	DMM	η_L	η_T	AMM	DMM	η_L	η_T	AMM	DMM
AlN	0.56	0.21	135	118	0.48	0.14	132	127	0.39	0.09	139	135
Al_2O_3	0.50	0.19	121	114	0.47	0.14	132	121	0.38	0.09	135	128
GaN	0.58	0.48	263	208	0.63	0.39	319	235	0.52	0.25	315	263
LiF	0.84	0.80	432	330	0.86	0.98	743	405	0.90	0.66	797	496
$LiNbO_3$	0.72	0.68	367	234	0.68	0.47	377	270	0.55	0.30	382	307
MgO	0.57	0.23	145	127	0.52	0.16	146	136	0.43	0.10	153	145
PET	0.53	0.68	356	472	0.21	0.35	261	641	0.25	0.42	481	905
Si	0.63	0.34	197	151	0.48	0.18	157	165	0.43	0.12	171	178
SiC (3C)	0.45	0.60	312	205	0.48	0.35	279	231	0.40	0.23	288	259
a-SiN	0.55	0.23	142	121	0.46	0.14	132	130	0.38	0.10	141	138
SiO_2	0.91	0.61	345	220	0.61	0.32	268	251	0.59	0.21	285	284
$SrTiO_3$	0.67	0.35	205	172	0.67	0.26	231	191	0.58	0.17	236	209

	NbN				TiN				WSi			
	η_L	η_T	AMM	DMM	η_L	η_T	AMM	DMM	η_L	η_T	AMM	DMM
AlN	0.68	0.48	123	94	0.64	0.46	142	100	0.30	0.29	100	105
Al_2O_3	0.65	0.50	127	91	0.57	0.45	137	97	0.30	0.32	107	101
GaN	0.96	0.93	228	142	0.94	0.98	286	157	0.45	0.74	225	169
LiF	0.86	0.90	219	190	0.96	0.94	278	218	0.72	0.76	253	241
$LiNbO_3$	0.88	0.84	206	154	0.98	0.95	281	172	0.44	0.63	196	186
MgO	0.74	0.54	138	99	0.68	0.51	157	106	0.33	0.34	113	111
PET	0.19	0.24	56	230	0.31	0.35	102	273	0.13	0.15	49	309
Si	0.66	0.52	133	113	0.69	0.58	174	122	0.32	0.31	104	129
SiC (3C)	0.69	0.73	177	141	0.62	0.89	250	156	0.38	0.51	160	167
a-SiN	0.64	0.47	121	95	0.61	0.47	143	102	0.29	0.28	96	107
SiO_2	0.62	0.68	164	148	0.81	0.83	243	164	0.37	0.45	146	177
$SrTiO_3$	0.90	0.91	222	124	0.98	0.83	250	136	0.44	0.58	184	144

Supplementary Table 5. Calculated values for the acoustic and diffuse mismatch models, for select superconductor-substrate pairs. For a given pair, η_L and η_T are the angle-averaged transmission coefficients for longitudinal and transverse phonon modes respectively. AMM and DMM refer to $\sigma_{ph}\epsilon_{AMM}$ and $\sigma_{ph}\epsilon_{DMM}$ which are the calculated values of the phonon black body thermal boundary conductance with units $Wm^{-2}K^{-4}$.

Supplementary Figure: calculated $\sigma_{ph}\epsilon_{AMM}$ for select substrate-superconductor pairs

In Fig. S2 we plot the AMM based thermal boundary conductance that is tabulated in Table S5.

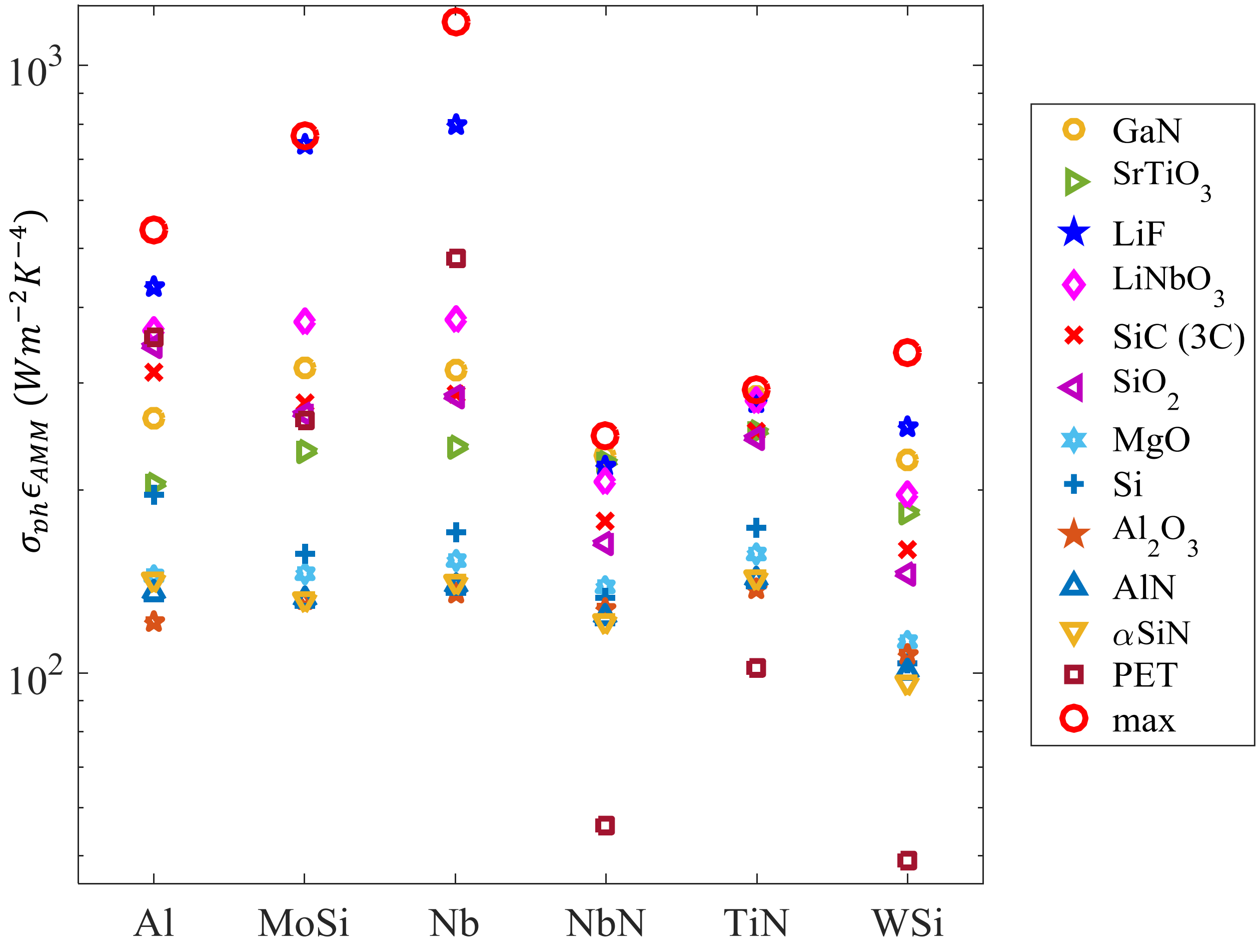

Supplementary Figure 2. Plot of calculated values of $\sigma_{ph}\epsilon_{AMM}$ for select superconductor-substrate pairs. The 'max' values correspond to hypothetical dielectrics that have identical acoustic properties as the corresponding metal layer and represent that theoretical maximum AMM based TBC achievable for that metal.

Supplementary Table: summary table for comparing β to $\sigma_{ph}\epsilon_{AMM}$ and $\sigma_{ph}\epsilon_{DMM}$

Table S6 provides all of the data necessary for carrying out the fitting documented in the main text, as well as the fitting results when $n = 4$ and when n is used a free parameter in the fit.

								n free			n = 4		beta, calc'd		
**Sample:**	**Sub.**	**d**	**w**	**l**	**R (12 K)**	**sq**	**Rs (12K)**	**Ths**	**n**	**beta_fit**	**Ths**	**beta_fit**	**AMM**	**DMM**	**η (2 K)**
		nm	nm	nm	kOhm	#	Ohm	K		W/(m^2 K^n)	K	W/(m^2 K^4)	W/(m^2 K^4)		nm
SPF221_BC	Al2O3	5	190	7106	20.5	37	547	8.5	3.7	202	8.4	106	128	91	162
SPF221_DC	Al2O3	5	190	7106	22.1	37	590	8.5	3.6	262	8.4	114	128	91	151
SPF221_CD	Al2O3	5	74	3330	25.7	45	570	8.4	3.5	390	8.3	133	128	91	142
SPF221_BD	Al2O3	5	74	3330	25.6	45	568	8.5	3.1	886	8.3	128	128	91	145
JLT000_7I	LiNbO3	5	100	65300	518.8	653	794	8.1	5.1	19	8.5	153	207	154	112
JLT000_7J	LiNbO3	5	100	65300	526.6	653	806	8.2	3.9	215	8.1	176	207	154	104
JLT000_7H	LiNbO3	5	100	65300	507.0	653	776	8.3	4.6	47	8.5	161	207	154	110
SPF746_B	PET	7	173	102070	439.8	590	745	9.0	2.2	1411	8.2	36	56	230	240
SPE995	Si	4A	133	75544	302.7	568	533	9.9	4.6	21	10.1	82	133	113	187
SPF028_B	Th.Ox.	5	88	48136	272.8	547	499	8.7	2.9	1644	8.4	166	163	147	136
SPF054_DB	Th.Ox.	5	400	8400	15.8	21	751	7.7	3.4	371	7.6	116	163	147	132
SPF054_DC	Th.Ox.	5	200	6000	33.2	30	1108	7.9	3.3	453	7.7	109	163	147	112
SPF054_DD	Th.Ox.	5	100	3600	36.5	36	1014	7.7	3.9	138	7.6	112	163	147	116
SPF054_CC	Th.Ox.	5B	200	6000	33.2	30	1107	7.7	3.8	217	7.7	152	163	147	95
SPF115_A1N	Th.Ox.	5C	50	4050	8.9	81	110	6.3	4.1	17	6.3	19	163	147	848
SPF115_A3S	Th.Ox.	5C	100	6400	7.7	64	120	5.5	4.8	8	5.7	29	163	147	660
SPF184_BB	SrTiO3	7	90	80000	338.1	889	380	9.1	4.6	66	9.4	234	222	124	131

A: NbN deposited with substrate heated to 840°C
B: Narrow track damaged with helium ions
C: 5 nm of Ti sputtered onto the top of the NbN, without breaking vacuum
D: temp where negative differential resistance region is no longer apparent.

Supplementary Table 6. Summary of inputs and results from fitting $I_{hs}(T_b)$ data. The data to the left of the boxed regions are inputs and identifying information. The boxed region gives the values of the fit parameters which result from fitting $I_{hs}(T_b)$. The calculated thermal boundary conductance for the given substrate is shown for convenience in the two rightmost columns. The highlighted rows correspond to $I_{hs}(T_b)$ data plotted in figure 2(a) and 2(b).

Supplementary Figures: all $I_{hs}(T_b)$ data and fits

Here we plot the hotspot current data collected for this work, along with the fits whose parameters are given in Table S6. The data for the NbN nanowire on polyethylene terephthalate (PET) is not shown, but will be presented and analyzed further elsewhere.

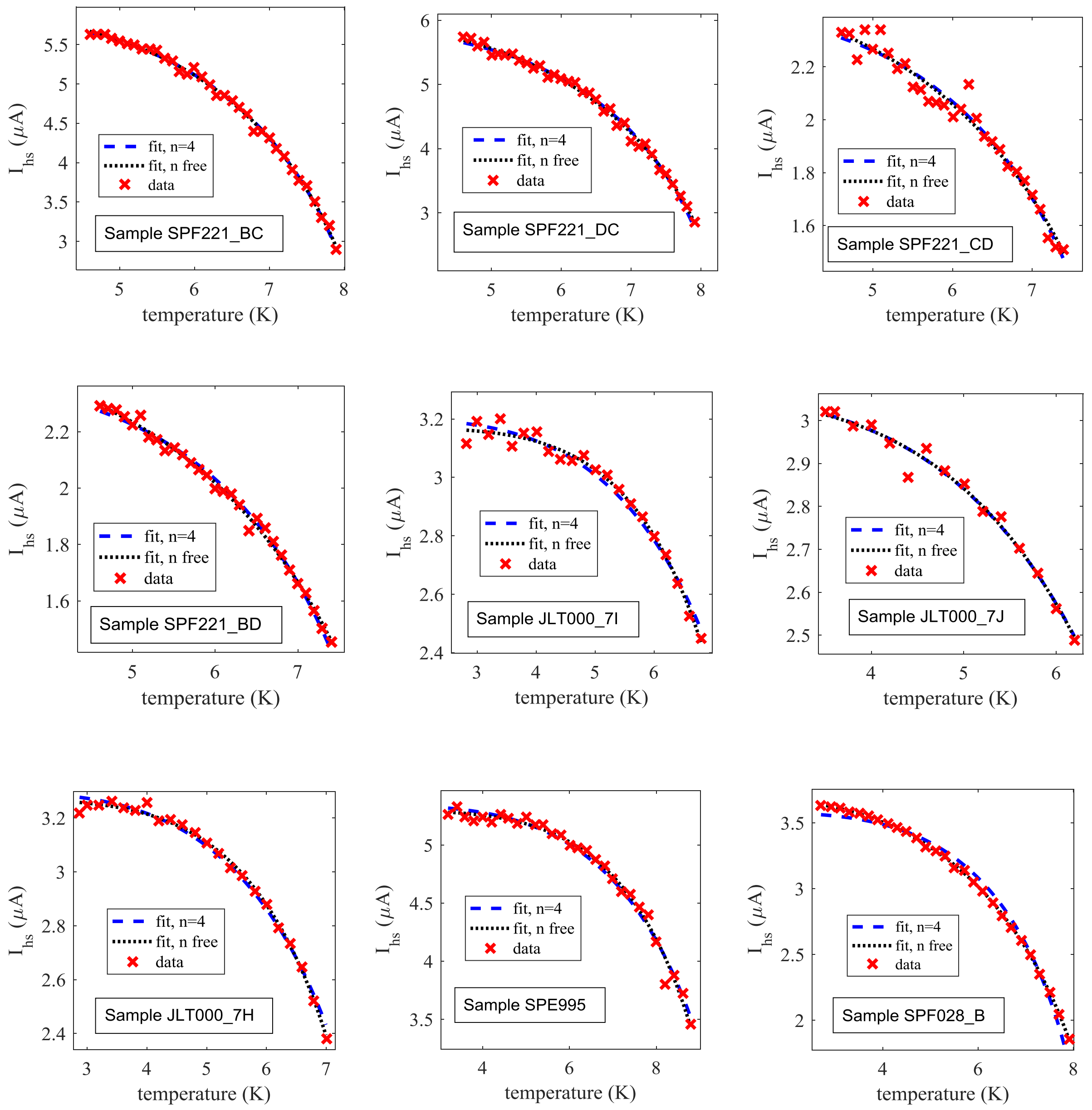

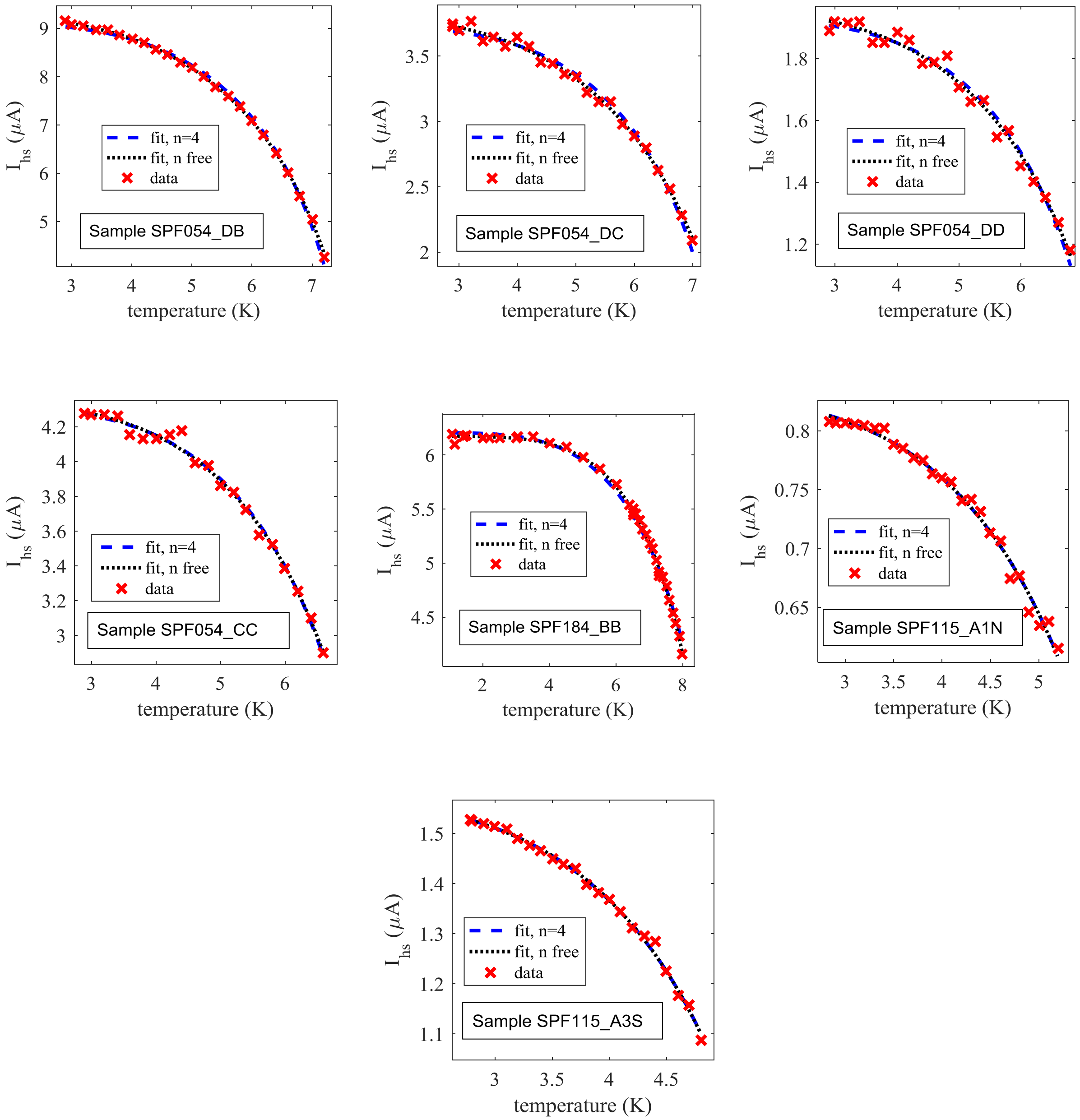

Supplementary Figure 3. Measured hotspot current vs bath temperature for all of the nanowires reported in this work, except for the NbN nanowire on PET which will be reported elsewhere. The measurement circuit used to gather this data is shown in the inset of Fig. 1(a).

Supplementary Note: our re-analysis of Kadin's data

Ref. [13] reports the linearized thermal resistance (R_{th}), extracted from measurements of the return (hotspot) current for NbN microwires on silicon substrates, which we re-analyze without linearization. R_{th} was extracted by fitting to the linearized Skocpol, Beasley, Tinkham (SBT) expression, $I_{hs}(T_b) \cong \sqrt{\frac{w^2(T_c-T_b)}{R_{th}R_s}}$, using R_{th} as a fitting parameter. Based on this, Johnson, Herr and Kadin concluded that the thermal resistance was approximately $8 \times 10^{-6}\ Km^2W^{-1}$ at 4.2 K. Using R_{th} extracted from figure 5 of their paper, we reconstructed the measured $I_{hs}(T_b)$ by inverting the given SBT equation, using device details provided in the text. We then fit the reconstituted $I_{hs}(T_b)$ using our phonon black body model. The extracted β is approximately 72% of the value expected from AMM. The linearized thermal resistance we would expect at 4.2 K based on the extracted β is approximately $35 \times 10^{-6}\ Km^2W^{-1}$, more than four times greater than what was extracted using the linearized SBT expression.

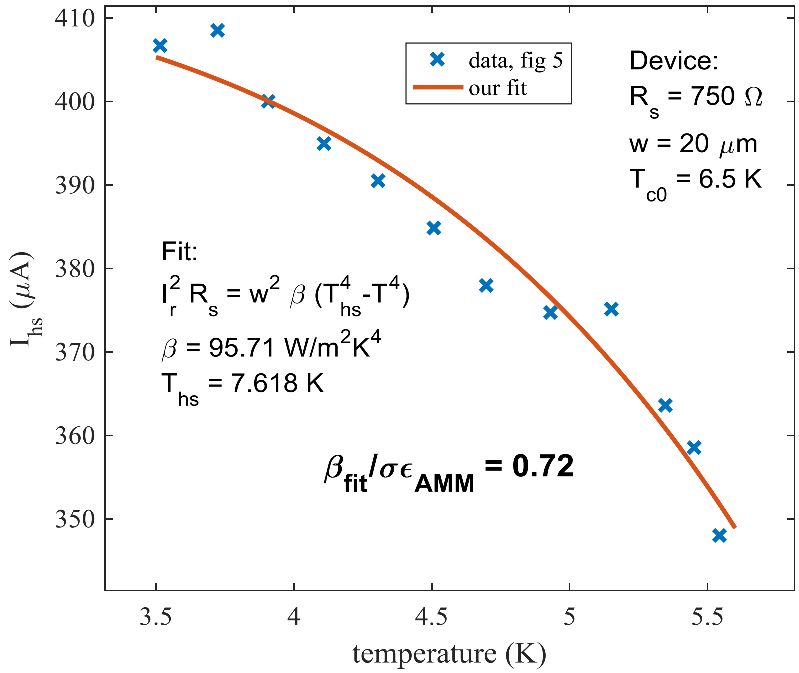

Supplementary Figure 4. Our re-analysis of data extracted from [13]. Data from Fig. 5 in ref [13] was extracted, and then converted to hotspot current data, using the device parameters and linearized SBT expression used by the authors (blue crosses). We then repeated our fitting process on this data, with $n = 4$, with the result plotted in orange. The extracted thermal boundary conductance is ~72% of what is expected by AMM for a NBN-Si interface. The linearized thermal boundary resistance consistent with this is more than four times higher than what was reported.

Supplementary Note: excluding the Nb-Ti bilayer devices

Here we explain our rationale for excluding two of our nanowire samples from parts of figure 2 in the main text. Despite finding that the fitting process works for these films, we are unable to acoustically model these films in the way that we can for all of the other samples.

Two of the nanowire devices (SPF115) measured for this work were made from NbN-Ti bilayer films. Each layer was approximately 5 nm thick. The NbN was deposited first, followed by the Ti, without breaking vacuum. Two 1 cm square SiO_2 substrates were deposited onto in the same sputtering process: one for fabrication and one for testing the unpatterned film.

Figure S3 compares the resistance vs temperature of the unpatterned film (blue, times 50) with that from the lithographed nanowires (red and magenta). The T_c of the unpatterned bilayer film is significantly less than the 8 to 9 K that we routinely measure for 5 nm thick NbN sputtered using the same process without Ti. We attribute the large drop in T_c of the NbN-Ti bilayer to the inverse proximity effect of the Ti on the NbN. We expect that the film of the patterned chip would have the same T_c prior to patterning, however it was not measured. After patterning, the nanowires display a T_c that is at least one K higher than the unpatterned film, with the narrower nanowire having two apparent transitions. This is the opposite of what is typically expected, as damage due to patterning typically reduces the wire T_c value with respect to the unpatterned film.

The increased T_c of the nanowires with respect to the unpatterned film is consistent with diffusion between the two layers creating some fraction of NbTiN, which has a higher T_c than NbN in bulk form. In studies of heating multi-layer films of TiN and NbN it has been shown that Ti diffuses into NbN, forming a layer of NbTiN while consuming the TiN layer [14]. While most of the processing of the chip is done near room temperature, some heating is expected during the reactive ion etching of the nanowires. A fixed heat flux at the wire edges during RIE would cause narrower structures to heat to higher temperatures, potentially causing more diffusion and thicker NbTiN layers. This could explain the double transition of the 50 nm nanowire.

Explaining the apparent increase in T_c requires assuming a layered structure of unknown composition. This makes it highly unsuitable for being described by the acoustic properties of single layer NbN as we were able to do for all other samples. While $I_{hs}(T_b)$ for these devices can be fit with the phonon black body model, suitable acoustic parameters are not known, and we therefore do not include the comparison to AMM and DMM in figure 2.

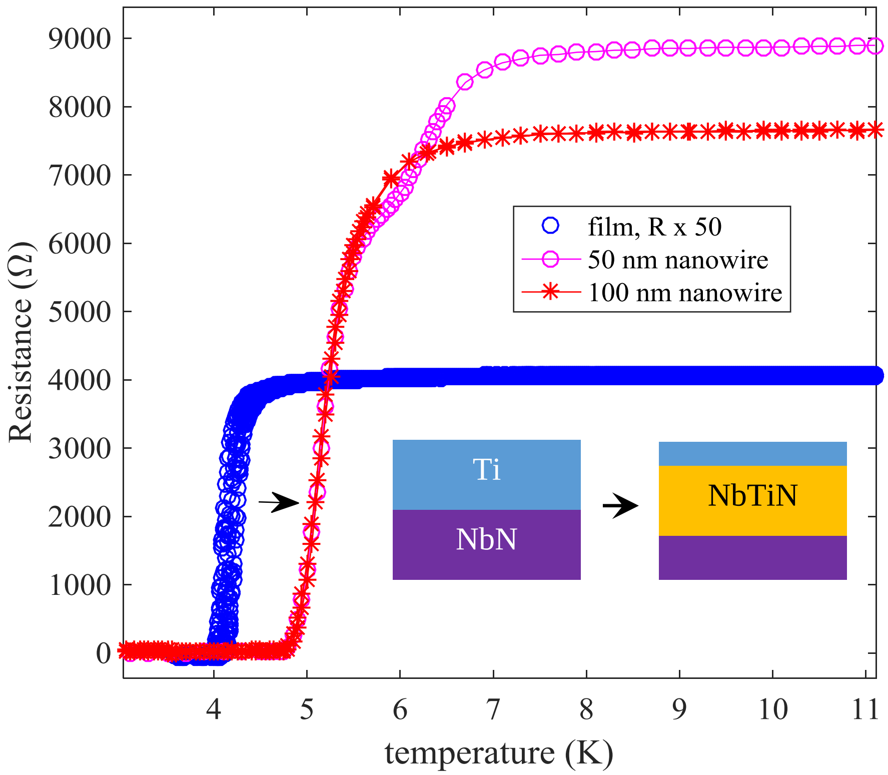

Supplementary Figure 5. Comparison of the resistive transition of an unpatterned NbN-Ti film (5 nm / 5nm. Shown times 50 in blue) and nanowires made from material deposited at the same time (red, magenta). The unpatterned film has a T_c near 4 K, which is four to five Kelvin lower than a 5 nm thick NbN film without Ti. The patterned nanowires have a higher T_c which we attribute to interdiffusion of NbN and Ti during fabrication, to create a higher T_c material. This is shown schematically in the inset. The double transition of the narrower wire may be evidence of greater levels of diffusion due to heating.

Supplementary References

[1] A. E. Dane *et al.*, "Bias sputtered NbN and superconducting nanowire devices," *Appl. Phys. Lett.*, vol. 111, no. 12, p. 122601, 2017.

[2] F. Najafi *et al.*, "Fabrication Process Yielding Saturated Nanowire Single-Photon

Detectors With 24-ps Jitter," *IEEE J. Sel. Top. Quantum Electron.*, vol. 21, no. 2, pp. 1–7, 2015.

[3] J. Bardeen, G. Rickayzen, and L. Teword, "Theory of the Thermal Conductivity of Superconductors*," *Phys. Rev.*, vol. 113, no. 4, 1959.

[4] S. B. Kaplan, C. C. Chi, D. N. Langenberg, J. J. Chang, S. Jafarey, and D. J. Scalapino, "Quasiparticle and phonon lifetimes in superconductors*," *Phys. Rev. B*, vol. 14, no. 11, pp. 4854–4872, 1976.

[5] D. Y. Vodolazov, "Single-Photon Detection by a Dirty Current-Carrying Superconducting Strip Based on the Kinetic-Equation Approach," *Phys. Rev. Appl.*, vol. 7, no. 3, pp. 1–19, 2017.

[6] M. Y. Kupriyanov and V. F. Lukichev, "Temperature Dependence of Pair-breaking Current in Superconductors," *Sov. J. Low Temp. Phys.*, vol. 6, no. 4, 1980.

[7] M. Sidorova *et al.*, "Electron energy relaxation in disordered superconducting NbN films," *Phys. Rev. B*, vol. 102, no. 5, p. 54501, 2020.

[8] E. T. Swartz and R. O. Pohl, "Thermal boundary resistance," *Rev. Mod. Phys.*, vol. 61, no. 3, pp. 605–668, 1989.

[9] J. Cheeke, H. Ettinger, and B. Hebral, "Analysis of heat transfer between solids at low temperatures," *Can. J. Phys.*, vol. 54, no. 17, pp. 1749–1771, 1976.

[10] S. B. Kaplan, "Acoustic matching of superconducting films to substrates," *J. Low Temp. Phys.*, vol. 37, no. 3–4, pp. 343–365, Nov. 1979.

[11] S. Krause *et al.*, "Reduction of Phonon Escape Time for NbN Hot Electron Bolometers by

Using GaN Buffer Layers," *IEEE Trans. Terahertz Sci. Technol.*, vol. 7, no. 1, pp. 1–7, 2017.

[12] M. V. Sidorova *et al.*, "Nonbolometric bottleneck in electron-phonon relaxation in ultrathin WSi films," *Phys. Rev. B*, vol. 97, no. 184512, 2018.

[13] M. W. Johnson, A. M. Herr, and A. M. Kadin, "Bolometric and nonbolometric infrared photoresponses in ultrathin superconducting NbN films," *J. Appl. Phys.*, vol. 79, no. 9, pp. 7069–7074, 1996.

[14] C. Engström *et al.*, "Interdiffusion studies of single crystal TiN/NbN superlattice thin films," *J. Vac. Sci. Technol. A Vacuum, Surfaces, Film.*, vol. 17, no. 5, pp. 2920–2927, 1999.
